# A pilot clinical skills coaching program to reimagine remediation: a cohort study

**DOI:** 10.12688/mep.19621.2

**Published:** 2023-07-13

**Authors:** Jean E. Klig, William M. Kettyle, Joshua M. Kosowsky, William R. Phillips, Jr., Susan E. Farrell, Edward M. Hundert, John L. Dalrymple, Mary Ellen J. Goldhamer

**Affiliations:** 1 Massachusetts General Hospital, Boston, Massachusetts, 02114, USA; 2 Harvard Medical School, Boston, Massachusetts, 02115, USA; 3 Brigham and Women’s Hospital, Boston, Massachusetts, 02115, USA; 4 Beth Israel Deaconess Medical Center and Harvard Medical School, Boston, Massachusetts, 02115, USA

**Keywords:** Remediation, coaching, clinical skills, medical student, undergraduate medical education, growth mindset, objective structured clinical exam (OSCE), strengths-based coaching

## Abstract

Background

New approaches are needed to improve and destigmatize remediation in undergraduate medical education (UME).  The COVID-19 pandemic magnified the need to support struggling learners to ensure competency and readiness for graduate medical education (GME).  Clinical skills (CS) coaching is an underutilized approach that may mitigate the stigma of remedial learning.

Methods

A six-month CS coaching pilot was conducted at Harvard Medical School (HMS) as a destigmatized remedial learning environment for clerkship and post-clerkship students identified as ‘at risk’ based on objective structured clinical examinations (OSCE).  The pilot entailed individual and group coaching with five faculty, direct bedside observation of CS, and standardized patient encounters with video review. Strengths-based coaching principles and appreciative inquiry were emphasized.

Results

Twenty-three students participated in the pilot: 14 clerkship students (cohort 1) and 9 post-clerkship students (cohort 2).  All clerkship students (cohort 1) demonstrated sustained improvement in CS across three OSCEs compared to baseline: at pilot close, at 6-months post pilot, and at 21-24 months post-pilot all currently graduating students (10/10, 100%) passed the summative OSCE, an HMS graduation requirement. All post-clerkship students (cohort 2) passed the HMS graduation OSCE (9/9,100%). Feedback survey results included clerkship students (9/14; 64%) and post-clerkship students (7/9; 78%); all respondents unanimously agreed that individual coaching was “impactful to my clinical learning and practice”. Faculty and leadership fully supported the pilot as a destigmatized and effective approach to remediation.

Conclusion

Remediation has an essential and growing role in medical schools.  CS coaching for remedial learning can reduce stigma, foster a growth mindset, and support sustained progress for ‘at risk’ early clerkship through final year students. An “implementation template” with suggested tools and timelines can be locally adapted to guide CS coaching for UME remediation. The CS coaching pilot model is feasible and can be generalized to many UME programs.

## Introduction

Remediation programs constitute a vital resource for medical students who are at risk of insufficiently advancing towards clinical competence milestones ^1– 4^. Yet remediation is commonly fraught with stigma that both limits its acceptance and impedes effectiveness ^5^. Discontinuation of the United States (U.S.) Medical Licensing Examination Clinical Skills (CS) Step 2 examination, which had a 5% failure rate ^6^, highlights the urgency for medical schools to identify and remediate ‘at risk’ students and certify clinical competence and readiness to advance to graduate medical education (GME) ^7^. A recent survey of American Medical Association (AMA) Accelerating Change in Medical Education consortium schools found that among 25 medical school coaching programs, only three programs focused on remediation and two programs focused on clinical skills development, the majority of programs focused on academic and professional development ^8^. Coaching programs less commonly focus on CS development or remediation in UME, though these are areas of growing research ^9, 10^. CS coaching offers a promising avenue to transform remediation as an explicitly learner-driven process that can address this gap and provide destigmatized clinical learning support in medical school ^8, 11, 12^. A coach can serve as a non-judgmental advocate who provides a positive environment where a struggling student is prompted to reflect on strengths, co-create meaningful learning, and build clear and achievable steps towards success ^11, 13^. Advances in remediation are essential, notably as the COVID-19 pandemic has magnified the need to support struggling learners and to ensure competency across the UME to GME continuum ^14, 15^. The aim of this pilot was to establish the feasibility of CS coaching as normalized remedial support that allowed students to simultaneously engage in medical school classes and clerkships while improving clinical skills.

## Methods

### Ethics

All studies that involve Harvard Medical School (HMS) students and/or teaching faculty as study subjects or analysis of existing data related to HMS students require the approval of the HMS Program in Medical Education (PME) Educational Scholarship Review Committee. The Harvard Longwood Campus IRB has given this committee authority to designate studies it deems quality improvement to be IRB exempt. The clinical skills coaching pilot met the criteria for a quality improvement project and was deemed IRB exempt and participant consent was waived as part of this exemption.

A six-month CS coaching pilot was developed at Harvard Medical School (HMS) to promote a destigmatized learning environment for clerkship and post-clerkship students identified as ‘at risk’ based on objective structured clinical examination (OSCE) performance. Students were identified as ‘at risk’ based on low OSCE performance and/or concerns on narrative evaluations. A ‘below passing’ OSCE performance was the lowest fifteenth percent of scores for the clerkship cohort (cohort 1). Two students in the clerkship cohort 1 with OSCE scores at the mean on pilot entry were identified based on narrative evaluations. [Table 1] The post-clerkship cohort (cohort two) was identified based scores below the passing threshold score of 68% on the HMS summative graduation OSCE. The 68% total passing score for the HMS summative graduation OSCE was calculated using a modified Hofstee method ^16^ that incorporates historical data of students’ performance and a faculty mean score based on expectations for minimum and maximum passing scores and percentages.

 The pilot entailed three overlapping phases: faculty development; co-production of student individualized learning plans (ILPs) coupled with group and individualized coaching; and post-pilot transition planning. Faculty development and coaching occurred from July to December 2019, and post-pilot longitudinal OSCE follow-up spanned two years (January 2020 to December 2021). A total of six CS focus areas corresponded to elements of the Association of American Medical Colleges (AAMC) Core Entrustable Professional Activities (EPAs) for Entering Residency: history-taking, physical examination, clinical reasoning, oral presentation, communication, and learning on a clinical team ^17^.

**Figure 1.**
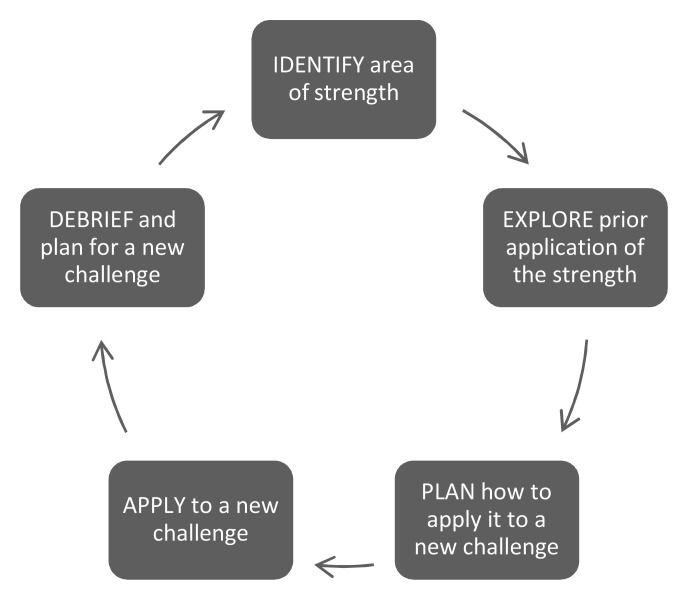
Strengths-based coaching cycle for the Clinical Skills (CS) pilot.

 A coaching team was identified by HMS leadership for their CS expertise and included five faculty from the core HMS OSCE faculty based on their diverse roles as experienced clinical educators, interest in coaching, and respective clinical training sites and disciplines. Faculty development focused on appreciative inquiry methods and a strengths-based coaching framework to engage each student in goal-based progress and support their development as master adaptive learners ( Figure 1) ^18– 20^. These coaching frameworks were chosen over models of behavior change to promote self-reflection, self-directed learning, and co-creation of learning goals. This framework aligned with the pilot’s CS coaching goals which aimed to engage students and promote lifelong learning strategies. Monthly faculty team conferences debriefed the coaching sessions’ successes and challenges to ensure all coaches engaged in productive and growth-empowered remediation. Coaches were compensated at the school’s usual teaching compensation rate.

**Figure 2.**
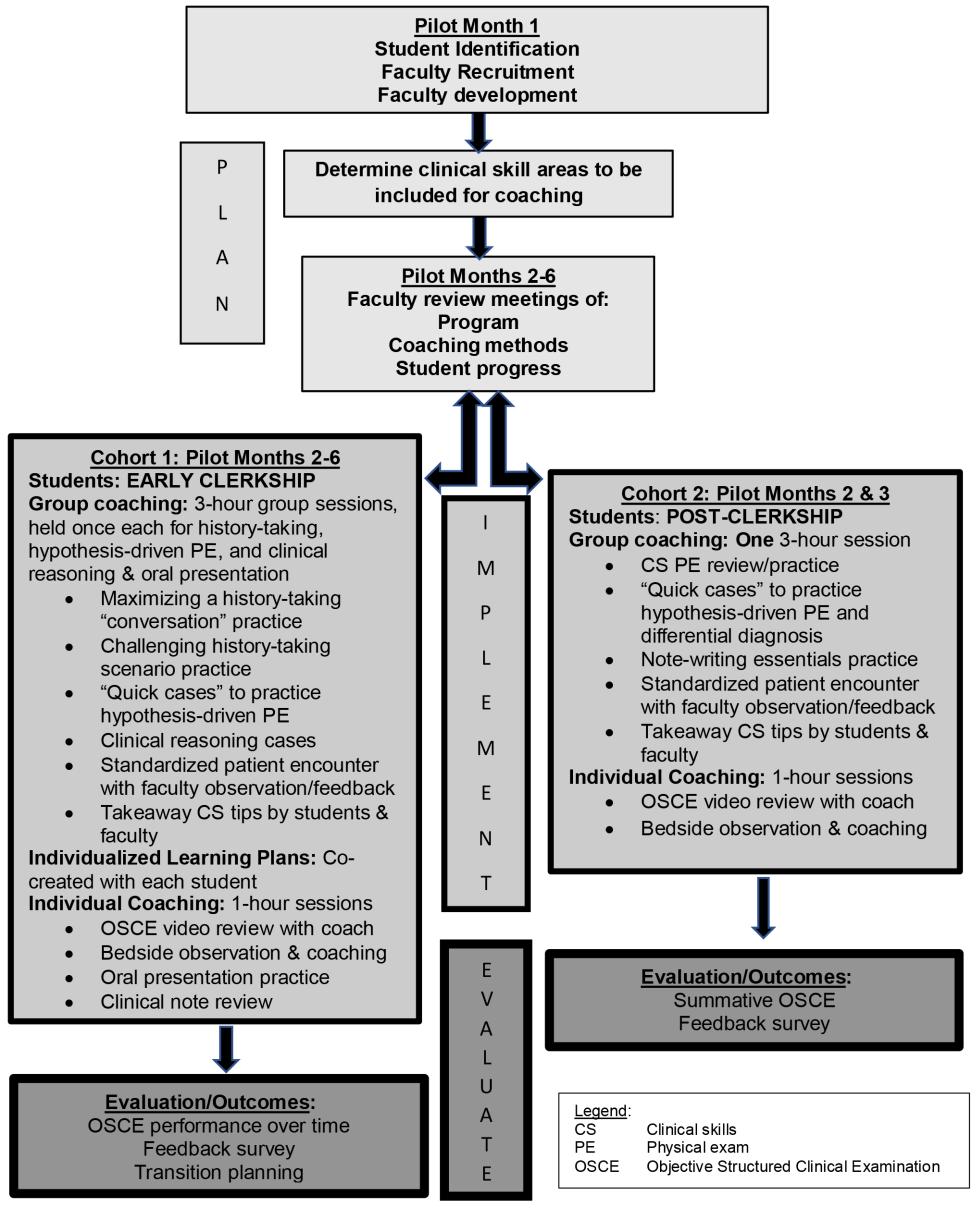
Implementation template for the coaching pilot.

A flow diagram details the planning, implementation, and evaluation of the CS coaching pilot ( Figure 2). CS learning support included a range of structured activities adaptable to identified student needs ( Figure 2). A coaching agreement upheld CS coaching as a non-evaluative confidential experience.

Clerkship students (cohort 1) attended three three-hour required group coaching sessions and could opt for individual coaching during clerkships. Individual sessions were optional to avoid overburdening students and to gauge student engagement in co-creating their learning. Assessment of the clerkship cohort’s CS included two formative OSCEs, each with three patient encounters incorporating immediate faculty feedback. These formative OSCEs were part of the routine HMS schedule at three and nine months after clerkship entry, corresponding to the close of the coaching pilot and six months post-pilot. A summative OSCE with nine patient encounters (and no formative feedback) required for graduation corresponded to two years post-pilot.

Each post-clerkship student attended one three-hour required group coaching session with optional individual coaching. Reassessment entailed a summative OSCE retake with new clinical case scenarios.

**Figure 3.**
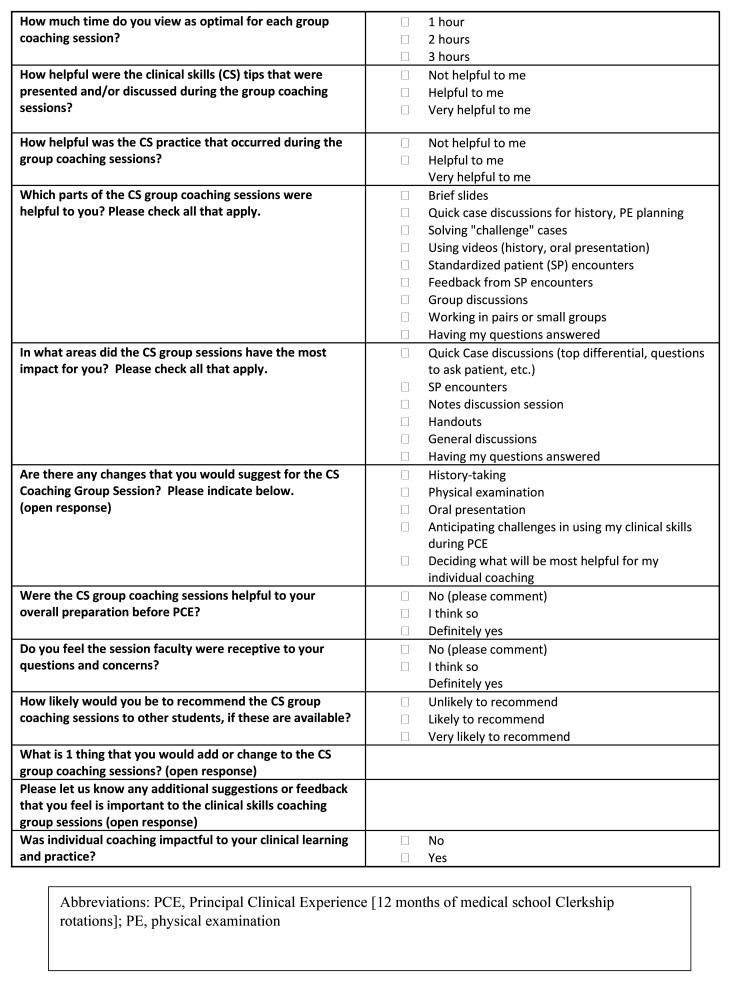
HMS Coaching Group Session Feedback Survey Questions [Cohort one, Clerkship Cohort].

**Figure 4.**
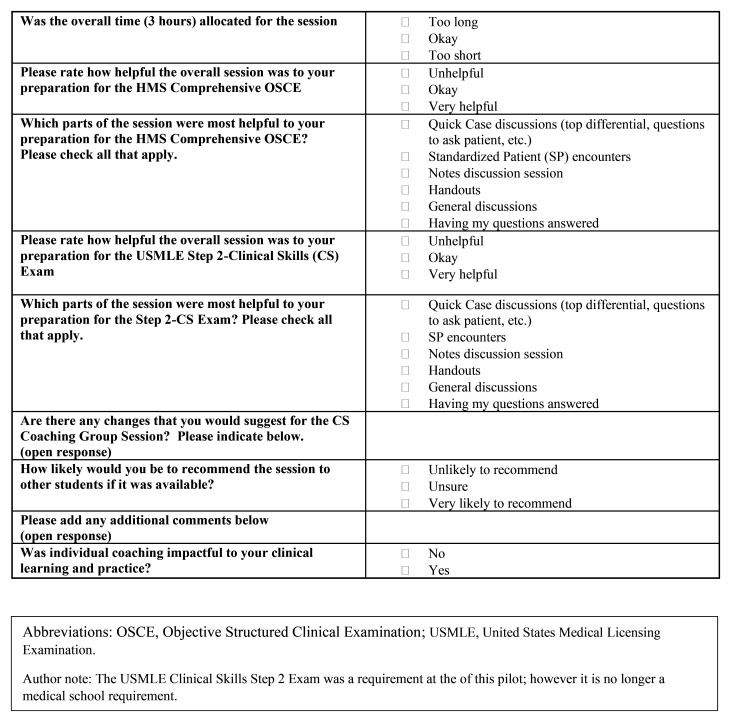
HMS Coaching Group Session Feedback Survey Questions [Cohort two, Post-clerkship Cohort].

Student engagement and feedback was monitored throughout the pilot as key indicators of creating a normalized learning support environment. This was done through frequent check-in meetings with students and iterative updates to student individualized learning goals made by program leadership and students working together, as well as student feedback surveys ( Figure 3 and Figure 4). The pilot closed at six-months with student transition planning which included the identification of student goals for clerkship rotations

## Results and analysis

A total of twenty-three students participated in the pilot: 14 clerkship students (cohort one) and 9 post-clerkship students (cohort two). There were 10 female and 13 male students across both cohorts. Students who were underrepresented in medicine made up less than 20% of both cohorts in the pilot. While group coaching sessions were required, optional hour-long individual coaching sessions were utilized by 57% (8/14) of clerkship students (24 total sessions, range two-seven sessions/student) and 44% (4/9) of post-clerkship students (six total sessions, range one-two sessions/student) during the pilot. Notably, 86% (12/14) of clerkship students participated in optional individual coaching during the combined pilot and post-pilot phases. Optional individual clerkship student (cohort 1) check-ins occurred in person, over email, and by phone and were offered monthly, with a range of 0–4 check-in meetings per clerkship student.

Pilot outcomes are summarized in Table 1. Five main outcomes include: OSCE assessment, student feedback on group and individual coaching, student feedback on coaching to normalize remediation, faculty satisfaction and feedback, and demonstrated feasibility as a program. 

**Table 1.** Outcomes by cohort and for the overall coaching pilot.

Outcomes	Cohort One: Clerkship Students N=14				Cohort Two: Post-Clerkship Students N=9	
**OSCE** **Results**	OSCE assessments *below* overall class mean				Summative OSCE retake: 100% pass (9/9)	
	**Pre-Clerkship**	**Clerkship ** **Month 3**	**Clerkship** **Month 9**	**Post-Clerkship ** **Summative**		
	***Pilot Entry***	***Pilot Close***	***Post-Pilot*** *(Six Months)*	***Post-Pilot*** *(21-24 Months)*		
	86% (12/14)	57% (8/14)	50% (7/14)	0% (0/10)		
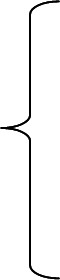	Responses 64% (9/14)				Responses 78% (7/9)	
	**“Most impactful areas”:**				**“Most helpful aspects”:**	
	History taking		78% (7/9)		OSCE + Coaching	86% (6/7)
	Hypothesis-driven PE practice		56% (5/9)		“Quick cases” for PE +DDx	71% (5/7)
	Oral presentation practice		56% (5/9)		Note writing essentials	71% (5/7)
	SP encounter		89% (8/9)			
	SP encounter feedback		78% (7/9)			
	“Was impactful to my clinical learning and practice”		100% (9/9)		100% (7/7)	
**Normalize ** **Remediation**	“Likely” or “Very Likely” to recommend coaching to peers		100% (9/9)		Value as “Helpful” to “Very Helpful”	100% (7/7)
**Coaching** **Faculty ** **satisfaction &** ** reflections**	• Created a safe space for student learning. • Created an opportunity for reflection on clinical skills gaps. • Responsive to rapid time frame, program and individual learning needs • Dynamic faculty engagement to continually align priorities. • Focused use of faculty time					
**Feasibility**	Pilot approved for a formal longitudinal learning support coaching program					

Abbreviations: Objective Structured Clinical Examination, (OSCE), Standardized Patient (SP), Clinical Skills (CS), Physical Exam (PE), Differential diagnosis (DDx)

**Figure 5.**
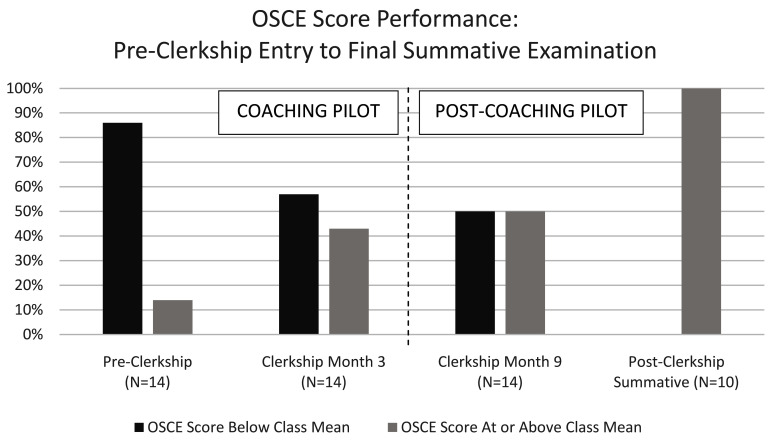
OSCE score performance for the coaching pilot clerkship cohort (cohort one). Pre-clerkship (pilot entry) to close of the six-month pilot (clerkship month three); Six-months post-pilot (clerkship month nine) to the post-clerkship summative graduation OSCE assessment (21–24 months post-pilot).

All clerkship students (cohort one) demonstrated improvement in CS from the baseline OSCE across two subsequent formative OSCE assessments ( Figure 5): at the close of the six-month pilot (corresponding to clerkship month three) and at six months post pilot (corresponding to clerkship month 9). Scores that were at or above the mean increased from 14% (2/14) on the pilot entry (pre-clerkship) OSCE, to 43% (6/14) at the close of the six-month pilot (clerkship month three), and 50% (7/14) six-months post pilot (clerkship month nine). Two years post pilot, all eligible clerkship (cohort one) students passed the summative HMS graduation OSCE (10/10, 100%). The remaining students (4/14, 29%) will be eligible for this OSCE prior to graduation after planned research time and/or concurrent degree programs. All post-clerkship students in cohort two (9/9, 100%) passed the summative OSCE retake.

A brief student feedback survey (cohorts one and two, Figure 3 and Figure 4 respectively) highlighted strengths of group and individual coaching ( Table 1). All clerkship students who responded to survey questions targeted at normalizing remediation (9/14; 64%) were “likely” to “very likely” to recommend CS coaching to peers, and all responding post-clerkship students (7/9; 78%) rated the value of CS coaching as “helpful” to “very helpful”. History-taking practice and standardized patient (SP) encounter with coaching feedback were identified as most impactful for the clerkship (cohort one) group sessions. OSCE-type SP practice with coaching were most impactful for the post-clerkship cohort two group sessions. Survey respondents (cohort one and two) unanimously agreed that individual coaching was “impactful to my clinical learning and practice”. 

Faculty and HMS leadership fully supported the pilot as a destigmatized and effective approach to remediation. Feasibility was demonstrated by student progress, sustained student and faculty engagement across and beyond the six-month pilot, and subsequent approval as the “HMS Clinical Learning Coaching Program” to provide longitudinal clinical learning support.

## Discussion and conclusions

Remediation has an essential and growing role in medical schools ^21^. This pilot demonstrates that a CS coaching program is a feasible and scalable way to reconceptualize remediation, mitigate perceived stigma, and support progress in CS development for ‘at risk’ medical students. CS coaching is an underutilized resource ^8^ and this six-month pilot provides “proof of concept” at one medical school with sustained CS improvement demonstrated by OSCE performance in clerkship students (cohort one) over two post-pilot years. The skills of reflective practice and self-directed learning were iteratively emphasized through appreciative inquiry and strengths-based coaching strategies, and it is likely the students applied these strategies to other aspects of their learning, resulting in sustained improvement in OSCE scores over two years.

This coaching pilot builds on existing work that addresses clinical skills remediation ^22^, notably for students identified as ‘at risk’ based on a multi-station summative OSCE ^23^ or for graduate medical education learners ^24^, by specifically designing coaching-based activities for two levels of undergraduate learners (clerkship students and post-clerkship students). It aims to further the learner-driven aspects of prior studies, in this case allowing targeted student remediation ^25^ to be guided by faculty through a dynamic and iterative co-creation process ^26^. The pilot contributes towards a standardized approach to active remediation, with trained faculty coaches to support struggling learners. Key aspects of a targeted remediation ^25^ were embedded within the coaching pilot, whereby the individual student performance, stated goals, and engagement in individual and group coaching sessions shaped a clear process that was locally available and integral to the standard Harvard Medical School curriculum. 

It is important to note that this pilot study did not enroll participants based on pre-defined inclusion or exclusion criteria other than participants’ demonstrated need for clinical skills remediation in medical school. All students identified as needing learning support were included in the pilot and the outcome of the pilot looked at quantitative clinical exam scores. This small feasibility education pilot study was not powered to look at sex and gender differences, though this can be considered in future studies that include larger cohorts.

A key lesson is that CS coaching reframed the learning process from a ‘gap’ or remedy-focused approach to one of guided self-directed learning and growth as master adaptive learners ^19^. Additional lessons include the value of appreciative inquiry to redirect individual student concerns about having “failed” ^27^. Strengths-based coaching strategies build towards improved CS and learning while fostering a growth mindset ^28, 29^. Group coaching allowed students to adjust to the pilot with their peers, while individual sessions at clerkship sites provided coaching in settings integral to daily clinical learning. The alignment of coaching with the medical school curriculum enabled students to simultaneously participate in classes and clerkships, thereby remaining on track with peers. Identifying clear endpoints and student transition planning is essential. Next steps include expansion of the program within the HMS curriculum ^30^ as a formal longitudinal learning support program with a focus on early identification and engagement of students during pre-clerkship training, to avoid simultaneous remediation of core CS during clerkship learning ^31^. A CS coaching “implementation template” ( Figure 2) with suggested activities and timelines can be locally adapted to guide remediation in UME. Further adaptations of the template are encouraged to collaboratively develop best practices in CS coaching across medical schools.

As a limitation, the small total numbers (n=23) in this feasibility “proof of concept” pilot reflect a specific cohort of ‘at risk’ learners for whom remediation was most critically needed. Nevertheless, the findings demonstrate the benefits of destigmatized remediation and offer a pathway to reimagine clinical skills learning support for larger cohorts of struggling or ‘at-risk’ students. A further limitation of the pilot was the exclusive use of OSCE assessment scores to evaluate student progress. This measure is a standardized metric by which all HMS students are evaluated, though future studies may include iterative AAMC EPA ratings, end-of-rotation evaluations, narrative comments, and other assessments for a holistic view of student progress. Finally, we were only able to complete two-year longitudinal follow-up for students in the clerkship cohort (cohort one), as the post-clerkship cohort (cohort two) students were in the final year of training at the time of the pilot.

It is important to note that the OSCEs are scored by both trained Standardized Patients (SPs) and HMS OSCE core educator faculty. SPs score the communication skills component of the OSCE only, using a 5-point Likert scale. SPs receive implicit bias training during their new hire orientation session and ongoing refresher training during each training session. HMS core educator faculty score the remainder of the OSCE, and checklists used to calculate an OSCE score are mostly based on a binary response (yes/no) to indicate presence or absence of an observed skill. These binary checklist scores are less likely to reflect implicit or unconscious bias of the evaluator. These biases, however, may occur in narrative evaluation comments, while selecting an AAMC EPA rating ^32^, and/or during feedback sessions. While there is ongoing faculty development aimed at detecting and eliminating unconscious and implicit bias in feedback sessions and on written evaluations, this training was not in place in 2019 at the time of the pilot. The authors appreciate the evidence in the literature that describes these biases ^32^ and recognize the importance of ongoing work to detect and extinguish unconscious and implicit bias in medical student assessment and feedback.

The CS coaching pilot demonstrates the feasibility of coaching to address the needs of ‘at risk’ learners in a destigmatized, supportive, and growth-oriented manner with measurable success. As the coaching interventions of the pilot may also be beneficial to all medical students, we plan to pilot optional student workshop sessions based on the three group sessions for clerkship students this year. CS coaching for remedial learning can offer a structured approach to promote competency and readiness for the UME to GME transition ^33– 35^.

## Data Availability

The datasets generated and analyzed during the current study are not publicly available in an open repository in accordance with the policies outlined in The Harvard Medical School Medical Education Student Handbook section on “Academic Information and Policies,” section 2.16 “ **Program Evaluation and Education Research”** ^36^ which states that “when reported, any research involving aggregated/de-identified student data will use only aggregate student information to maintain strict confidentiality.” ^36^ Access to the data is available from the corresponding author, Dr. Klig, upon reasonable request including those who request to examine the aggregated survey data.
