# Supplementary material for: A pilot clinical skills coaching program to reimagine remediation: a cohort study
Source: MedEdPublish (2016). 2023 Jul 13;13:29. Originally published 2023 May 18. [Version 2] doi: 10.12688/mep.19621.2 (PMC10477753; doi:10.12688/mep.19621.2)
Supplement: Peer review history

# Peer review: Reviewer response for version 2

**DOI:** 10.21956/mep.21127.r34122

**Published:** 2023-09-04

**Authors:** Kalman Winston

**Affiliations:** 1 Department of Public Health and Primary Care, University of Cambridge, Cambridge, UK

Happy to approve this now.

Have any limitations of the research been acknowledged?

Yes

Is the study design appropriate and does the work have academic merit?

Yes

Is the work clearly and accurately presented and does it cite the current literature?

Yes

If applicable, is the statistical analysis and its interpretation appropriate?

Not applicable

Are all the source data underlying the results available to ensure full reproducibility?

No

Are the conclusions drawn adequately supported by the results?

Yes

Are sufficient details of methods and analysis provided to allow replication by others?

Partly

Reviewer Expertise:

NA

I confirm that I have read this submission and believe that I have an appropriate level of expertise to confirm that it is of an acceptable scientific standard.

# Peer review: Reviewer response for version 2

**DOI:** 10.21956/mep.21127.r34121

**Published:** 2023-07-31

**Authors:** Calvin Chou

**Affiliations:** 1 Department of Medicine, University of California and Veterans Affairs Healthcare System, San Francisco, CA, USA

Deepest appreciation to the authors for addressing the comments with such detail. One final item: for query #6, about the clarification phase of remediation, I think it would be important to state explicitly that individual meetings were held with each student to more deeply investigate other potential influences on their performance. Fully agree that for confidentiality, specifics of the conversations need not be revealed.

Have any limitations of the research been acknowledged?

Partly

Is the study design appropriate and does the work have academic merit?

Yes

Is the work clearly and accurately presented and does it cite the current literature?

Partly

If applicable, is the statistical analysis and its interpretation appropriate?

Not applicable

Are all the source data underlying the results available to ensure full reproducibility?

Partly

Are the conclusions drawn adequately supported by the results?

Yes

Are sufficient details of methods and analysis provided to allow replication by others?

Yes

Reviewer Expertise:

Feedback and remediation in health professions education

I confirm that I have read this submission and believe that I have an appropriate level of expertise to confirm that it is of an acceptable scientific standard.

# Peer review: Reviewer response for version 1

**DOI:** 10.21956/mep.21020.r33391

**Published:** 2023-06-09

**Authors:** Calvin Chou

**Affiliations:** 1 Department of Medicine, University of California and Veterans Affairs Healthcare System, San Francisco, CA, USA

**Update Note from MededPublish – 21st July 2023**

**This report has been updated to include a Competing Interest statement**

 I commend the authors on an important description of a remediation coaching program that is thoughtful, methodically designed and implemented, and assessed, with high satisfaction by the learners. This description adds to the literature about effective means to support learners who struggle with clinical skills development.

 Several areas raised questions for me.

 Introduction: needs a stronger aim statement: I think you are saying that since currently existing coaching programs in US medical schools focus less on clinical skills development or remediation, you set forth to determine the efficacy of a coaching program for students who struggle with clinical skills. Can you also differentiate between the coaching program you developed and descriptions of others (for example, Parsons et al, South Med J 2022 ^1^; Guerrasio et al, Acad Med 2019 ^2^; Chang et al, Med Education 2008 ^3^)?

 It seems that the “Ethics” subheading in the Methods section is extraneous.

 The development of the coaching program itself seems robust, and using an appreciative, strengths-based, and master adaptive framework is key; the authors could additionally invoke literature describing the optimal components of such a program (eg, Boileau et al, Adv Med Educ Pract 2017 ^4^).

 Figure 2 seems straightforward; using Steinert’s framework (Med Teach 2013 ^5^), one step that seems to be a bit underemphasized is clarification - after identification, clarifying the students’ needs: many times, students are subject to forces outside school that play outsized influences on a student’s performance.

 In terms of demographics: what was the proportion of underrepresented or minoritized learners, relative to the classes as a whole? How much of the nonpassing rate for students may have been because of implicit bias in evaluation by SPs? The group interventions that the authors use do not address these sadly common and more systemic influences.

 How many individual coaching sessions were set up for each student? This would start to address the amount of faculty time and effort it takes to shepherd students through processes like this.

 How similar were the formative OSCEs and the summative OSCE? If the formative ones looked very similar to the summative one (“teaching to the test”), then the impressive results of passing could weaken.

 I’m confused by the top panel in Table 1. If all students were selected on the basis of not passing formative or summative OSCEs, shouldn’t the pilot entry number be 100%? Or were pre-clerkship evolutions of other types taken into consideration?

 Finally, the results might imply a prevention strategy: to fully destigmatize learners from being labeled as being in remediation, perhaps might you consider adapting the three group interventions for the clerkship students into practice sessions for all medical students?

Have any limitations of the research been acknowledged?

Partly

Is the study design appropriate and does the work have academic merit?

Yes

Is the work clearly and accurately presented and does it cite the current literature?

Partly

If applicable, is the statistical analysis and its interpretation appropriate?

Not applicable

Are all the source data underlying the results available to ensure full reproducibility?

Partly

Are the conclusions drawn adequately supported by the results?

Yes

Are sufficient details of methods and analysis provided to allow replication by others?

Yes

Reviewer Expertise:

feedback and remediation in health professions education

I confirm that I have read this submission and believe that I have an appropriate level of expertise to confirm that it is of an acceptable scientific standard, however I have significant reservations, as outlined above.

## References

[1] : Characterization of Clinical Skills Remediation: A National Survey of Medical Schools. *Southern Medical Journal* .2022;115(3) : 10.14423/SMJ.0000000000001361 202-207 10.14423/SMJ.0000000000001361 35237839

[2] : The Evolution of Resident Remedial Teaching at One Institution. *Acad Med* .2019;94(12) : 10.1097/ACM.0000000000002894 1891-1894 10.1097/ACM.0000000000002894 31348065

[3] : Clinical skills remedial training for medical students. *Med Educ* .2008;42(11) : 10.1111/j.1365-2923.2008.03191.x 1118-9 10.1111/j.1365-2923.2008.03191.x 18991999

[4] : Is there a way for clinical teachers to assist struggling learners? A synthetic review of the literature. *Adv Med Educ Pract* .2017;8: 10.2147/AMEP.S123410 89-97 10.2147/AMEP.S123410 28176916PMC5260943

[5] : The "problem" learner: whose problem is it? AMEE Guide No. 76. *Med Teach* .2013;35(4) : 10.3109/0142159X.2013.774082 e1035-45 10.3109/0142159X.2013.774082 23496125

# Response

**Published:** 2023-07-01

**Authors:** Mary Ellen J. Goldhamer, MD MPH

**Affiliations:** Department of Medicine, Harvard Medical School and Massachusetts General Hospital, Boston, Massachusetts, USA

 Dear Dr. Chou, Thank you for your thoughtful and timely review of our manuscript. Your suggestions and literature references have been incorporated and strengthen our “Manuscript, Version 2”. We anticipate the updated “Version 2” manuscript will be circulated for your review in the next 1-2 weeks by the editorial staff.  We hope you will have time to re-review “Manuscript, Version 2” which accompanies our point-by-point responses to your queries below.  We thank you in advance for reconsidering our paper for publication.   Sincerely, Jean E. Klig, MD and Mary Ellen J. Goldhamer, MD MPH ( *on behalf of our author group*) **Reviewer query #1** I commend the authors on an important description of a remediation coaching program that is thoughtful, methodically designed and implemented, and assessed, with high satisfaction by the learners. This description adds to the literature about effective means to support learners who struggle with clinical skills development. **Author response #1** **: Thank you.**

   Several areas raised questions for me. **Reviewer Query #2** **Introduction: needs a stronger aim statement:** I think you are saying that since currently existing coaching programs in US medical schools focus less on clinical skills development or remediation, you set forth to determine the efficacy of a coaching program for students who struggle with clinical skills. **Author response #2: **Thank you for noting the need to highlight the aim of the pilot earlier in the manuscript. The aim statement was not modified ( underlined below) but was moved from the “Methods” section to the end of the “Introduction” section.  We have included some additional text from the Introduction here for context: *“A recent survey of American Medical Association (AMA) Accelerating Change in Medical Education consortium schools found that among 25 medical school coaching programs, only three programs focused on remediation and two programs focused on clinical skills development, the majority of programs focused on academic and professional development. Coaching programs less commonly focus on CS development or remediation in UME, though these are areas of growing research… The aim of this pilot was to establish the feasibility of CS (clinical skills) coaching as normalized remedial support that allowed students to simultaneously engage in medical school classes and clerkships while improving clinical skills.”* **Reviewer Query #3: Can you also differentiate between the coaching program you developed and descriptions of others (for example, Parsons et al, South Med J 2022** **^1^** **; Guerrasio et al, Acad Med 2019** **^2^** **; Chang et al, Med Education 2008** **^3^** **)?** ***Author response #3: *** Thank you for this query and for suggesting relevant literature citations aimed at strengthening our manuscript, which have been incorporated as noted below.   The following paragraph and literature references have been added to “Manuscript Version 2”, “Discussion and conclusions” section, “ *The coaching pilot builds on existing work that addresses clinical skills remediation (Parsons A, Warburton, K et al here), notably for students identified as ‘at risk’ based on a multi-station summative OSCE (Chang, Chou, Hauer) or for graduate medical education learners (Guerrasio, Brooks, Rumack), by specifically designing coaching-based activities for two levels of undergraduate learners (clerkship students and post-clerkship students).  It aims to further the learner-driven aspects of prior studies, in this case allowing targeted student remediation (Boileau, St-Onge, Audetat) to be guided by faculty through a dynamic and iterative co-creation process (Steinert Y). The pilot contributes towards a standardized approach to active remediation, with trained faculty coaches to support struggling learners.* *Key aspects of a targeted remediation (insert Boileau et al) were embedded within the coaching pilot, whereby the individual student performance, stated goals, and engagement in individual and group coaching sessions shaped a clear process that was locally available and integral to the standard Harvard Medical School curriculum.”* Author note: These additional full references will be incorporated into the manuscript by the MedEdPublish editorial staff per “Manuscript updated version guidelines”. References:

1. Parsons A, Warburton K, Martindale J, et al.: Characterization of Clinical Skills Remediation: A National Survey of Medical Schools. *Southern Medical Journal. *2022; **115** (3): 202-207 | Publisher Full Text
2. Chang, A., Chou, C.L. and Hauer, K.E. (2008), Clinical skills remedial training for medical students. Medical Education, 42: 1118-1119. https://doi.org/10.1111/j.1365-2923.2008.03191.x
3. Guerrasio, Jeannette MD; Brooks, Elizabeth PhD; Rumack, Carol M. MD; Aagaard, Eva M. MD. The Evolution of Resident Remedial Teaching at One Institution. Academic Medicine 94(12):p 1891-1894, December 2019. | DOI: 10.1097/ACM.0000000000002894
4. Boileau E, St-Onge C, Audétat M-C. Is there a way for clinical teachers to assist struggling learners? A synthetic review of the literature. *Adv Med Educ Pract*. 2017;8:89-97, https://doi.org/10.2147/AMEP.S123410

5. Yvonne Steinert (2013) The “problem” learner: Whose problem is it? AMEE Guide No. 76, Medical Teacher, 35:4, e1035-e1045, DOI: 10.3109/0142159X.2013.774082 **Reviewer Query #4**

**It seems that the “Ethics” subheading in the Methods section is extraneous.** **Author response #4:**  The “Ethics” sub-heading in the Methods section is required by the MedEdPublish Journal as it alerts readers to the section that describes the Institutional Review Board (IRB) process. **Reviewer Query #5:**

 The development of the coaching program itself seems robust, and using an appreciative, strengths-based, and master adaptive framework is key; the authors could additionally invoke literature describing the optimal components of such a program (eg, Boileau et al, Adv Med Educ Pract 2017 ^4^ ).

**Author response #5:**

**The following was added to the “Discussion and conclusions” section**: “Key aspects of a targeted remediation (insert Boileau et al) were embedded within the coaching pilot, whereby the individual student performance, stated goals, and engagement in individual and group coaching sessions shaped a clear process that was locally available and integral to the standard Harvard Medical School curriculum.” **Reviewer Query #6** Figure 2 seems straightforward; using Steinert’s framework (Med Teach 2013 ^5^ ), one step that seems to be a bit underemphasized is clarification - after identification, clarifying the students’ needs: many times, students are subject to forces outside school that play outsized influences on a student’s performance. **Author response #6**:  Thank you for these important queries. During individual 1:1 meetings with the coaching program director, students were encouraged to share experiences that may impact their learning and where possible, solutions were sought and implemented.  As these experiences and solutions are confidential and unique to each student, these discussions and interventions are not described in the manuscript. **Reviewer Query #7** In terms of demographics: what was the proportion of underrepresented or minoritized learners, relative to the classes as a whole? **Author response** **# 7: **The following statement has been added under “Results and analysis”, paragraph 1: “ *Students who were underrepresented in medicine made up less than 20% of both cohorts in the pilot”.* **Reviewer Query #8: **How much of the non-passing rate for students may have been because of implicit bias in evaluation by SPs? The group interventions that the authors use do not address these sadly common and more systemic influences. **Author response** **#8: **Thank you for this important raising this important question. The OSCEs are scored by both standardized patients and trained core educator faculty. Standardized patients (SPs) rate communication skills only and ratings are on a 5-point Likert scale. All SPs have extensive training on implicit bias as part of their 2-hour new hire orientation training with ongoing refresher training during each training session. While it is possible for implicit or unconscious bias to impact SP scores, these important steps have been taken to guard against it. Importantly, students are rarely, if ever, identified for formal remediation due to poor communication skills.   Harvard Medical School OSCE core educator faculty score all other aspects of each case on OSCE checklists that are mostly based on a binary response (yes/no) to indicate presence or absence of an observable skill. Thus, the checklists with binary ratings from observed student OSCEs are less likely to reflect implicit or unconscious bias as the skill was either observed or not observed.   Unconscious or implicit bias may occur in narrative evaluation comments and/or during feedback sessions. While there is currently ongoing faculty development aimed at detecting and eliminating unconscious/implicit bias in feedback sessions and on written evaluations, this training was not in place in 2019 at the time of the pilot. The authors appreciate the evidence in the literature that describes these biases and recognizes the importance of ongoing work to detect and extinguish unconscious and implicit bias in medical student assessment and feedback.   The following paragraph was added to the “Discussions and conclusions” section of paragraph 6: *“* *It is important to note that the OSCEs are scored by both Standardized Patients (SPs) and trained HMS OSCE core educator faculty.  SPs score the communication skills component of the OSCE only, using a 5-point Likert scale. SPs receive implicit bias training during their new hire orientation session and ongoing refresher training during each training session. HMS core educator faculty score the remainder of the OSCE, and the majority of the checklists used to calculate an OSCE score are based on a binary response (yes/no) to indicate presence or absence of an observed skill.  These binary checklist scores are less likely to reflect implicit or unconscious bias of the evaluator. *These biases, however, may occur in narrative evaluation comments, while selecting an AAMC EPA rating [insert Hauer, Park, Bullock reference], and/or during feedback sessions. While there is currently ongoing faculty development aimed at detecting and eliminating unconscious and implicit bias in feedback sessions and on written evaluations, this training was not in place in 2019 at the time of the pilot. The authors appreciate the evidence in the literature that describes these biases [Hauer, Park, Bullock, et al] and recognizes the importance of ongoing work to detect and extinguish unconscious and implicit bias in medical student assessment and feedback.”   Hauer Karen E. MD, PhD; Park Yoon Soo PhD; Bullock Justin L. MD, MPH; Tekian Ara PhD MHPE Title: “My Assessments Are Biased!” Measurement and Sociocultural Approaches to Achieve Fairness in Assessment in Medical Education, Academic Medicine: Journal of the Association of American Medical Colleges  DOI: 10.1097/ACM.0000000000005245   

**Reviewer Query #9** How many individual coaching sessions were set up for each student? This would start to address the amount of faculty time and effort it takes to shepherd students through processes like this. **Author response: #9: **The authors agree it is important to track faculty time dedicated to remediation.  Under “Results and Analysis” we included **: **“ *While group coaching sessions were required, optional hour-long individual coaching sessions were utilized by 57% (8/14) of clerkship students ( **24 total sessions, range two-seven sessions/student**) and 44% (4/9) of post-clerkship students ( **six total sessions, range one-two sessions/student**) during the pilot.” *Updates to the manuscript were not made as this information was included in “Manuscript Version 1”.

**Reviewer Query #10** How similar were the formative OSCEs and the summative OSCE? If the formative ones looked very similar to the summative one (“teaching to the test”), then the impressive results of passing could weaken. **Author response #10: **Students in the remediation coaching program were assessed on the same formative and summative OSCE exams as students who did not participate in the program on the same regularly scheduled intervals. The summative end of medical school comprehensive OSCE examination broadly examines skills all graduating medical students should achieve and is a Harvard Medical School graduation requirement. Coaching sessions targeted clinical skills essential for all graduating students and did not “teach to the test”. The Methods section, paragraph 6, was updated to include the following statement: “ *Assessment of the clerkship cohort’s CS included two formative OSCEs, each with three patient encounters incorporating immediate faculty feedback. These formative OSCEs were part of the routine HMS schedule at three and nine months after clerkship entry, corresponding to the close of the coaching pilot and six months post-pilot. A summative OSCE with nine patient encounters (and no formative feedback) required for graduation corresponded to two years post-pilot.”* **Reviewer Query #11**

 I’m confused by the top panel in Table 1. If all students were selected on the basis of not passing formative or summative OSCEs, shouldn’t the pilot entry number be 100%? Or were pre-clerkship evaluations of other types taken into consideration? **Author response #11:** Students in the clerkship cohort (cohort 1) were identified as ‘at risk’ based on either low formative OSCE performance compared to the class or concerns on narrative evaluations. Two students in this cohort (14%) with OSCE scores at the class mean on pilot entry were identified based on narrative evaluations.    The revised “Manuscript Version 2” Methods section, paragraph 2, statement **has been revised for clarification from:** “ *The clerkship student cohort (cohort one) was identified based on below passing pre-clerkship OSCE scores **and** evaluations*” **to now state:** “ *Students were identified as ‘at risk’ based on low OSCE performance and/or concerns on narrative evaluations. A ‘below passing’ OSCE performance was the lowest fifteenth percent of scores for the clerkship cohort (cohort 1). Two students in the clerkship cohort 1 with OSCE scores at the mean on pilot entry were identified based on narrative evaluations. [Table 1] The post-clerkship cohort (cohort two) was identified based scores below the passing threshold score of 68% on the HMS summative graduation OSCE.* *The 68% total passing score for the HMS summative graduation OSCE was calculated using a modified Hofstee method [* *Burr, S.A., Whittle, J., Fairclough, L.C.* *et al* *.] that incorporates historical data of students’ performance and a faculty mean score based on expectations for minimum and maximum passing scores and percentages*.”An earlier reference to Table 1 has been added.

   Reference: Burr, S.A., Whittle, J., Fairclough, L.C. *et al.* Modifying Hofstee standard setting for assessments that vary in difficulty, and to determine boundaries for different levels of achievement. *BMC Med Educ* **16**, 34 (2016). https://doi.org/10.1186/s12909-016-0555-y 

**Reviewer Query #12** Finally, the results might imply a prevention strategy: to fully destigmatize learners from being labeled as being in remediation, perhaps might you consider adapting the three group interventions for the clerkship students into practice sessions for all medical students? **Author response #12**: Thank you. The authors agree that ideally the interventions described would likely be beneficial for all medical students and we plan to pilot optional student workshop sessions based on the three group interventions for the clerkship students this coming academic year.   The following text was added to the last paragraph of the Discussion and conclusions section, “ *As the coaching interventions of the pilot may also be beneficial to all medical students, we plan to pilot optional student workshop sessions based on the three group sessions for clerkship students this year*.”      1. Parsons A, Warburton K, Martindale J, et al.: Characterization of Clinical Skills Remediation: A National Survey of Medical Schools. *Southern Medical Journal. *2022; **115** (3): 202-207 | Publisher Full Text 2. Guerrasio J, Brooks E, Rumack CM, et al.: The Evolution of Resident Remedial Teaching at One Institution. *Acad Med. *2019; **94** (12): 1891-1894 PubMed Abstract | Publisher Full Text 3. Chang A, Chou CL, Hauer KE: Clinical skills remedial training for medical students. *Med Educ. *2008; **42** (11): 1118-9 PubMed Abstract | Publisher Full Text 4. Boileau E, St-Onge C, Audétat MC: Is there a way for clinical teachers to assist struggling learners? A synthetic review of the literature. *Adv Med Educ Pract. *2017; **8** 89-97 PubMed Abstract | Publisher Full Text 5. Steinert Y: The "problem" learner: whose problem is it? AMEE Guide No. 76. *Med Teach. *2013; **35** (4): e1035-45 PubMed Abstract | Publisher Full Text

# Peer review: Reviewer response for version 1

**DOI:** 10.21956/mep.21020.r33390

**Published:** 2023-05-22

**Authors:** Kalman Winston

**Affiliations:** 1 Department of Public Health and Primary Care, University of Cambridge, Cambridge, UK

This study appears to be clearly presented, cites current literature, and showcases an interesting and valuable approach to remediation. The authors draw on a number of important principles, including a blend of both group and individual approaches, using a strength based appreciative enquiry coaching method that helpfully avoids a deficit model and thus appears to reduce the stigma of remediation.

 The paper could be improved with clarification of a few points:

 You state that students were identified as ‘at risk’ based on OSCE performance of ‘below passing’. What exactly does this entail? You seem to be reporting results relative to the class mean. Are you saying everyone below the mean is not passing? Obviously not, but it is unclear. It would be helpful to offer some rationale for your choice of coaching model: why strengths-based framework, as opposed to other options? You state that “Each student attended a three-hour required group coaching session”, but figure 2 seems to be imply multiple group sessions. Can you clarify this discrepancy? What was the rationale for the decision to make the individual sessions optional? There were “frequent check-in meetings”. How many of these per student? How frequent? Optional or required? Individual or group? Figure 5 suggests 14% of cohort one had pre-clerkship OSCE performance at or above class mean, and yet earlier you stated that entry to the pilot required a below passing score. How is this discrepancy explained? Or am I misunderstanding the data, in which case more clarity may be needed, perhaps? The sustained improvement beyond the pilot is impressive – readers might be interested to hear why you think this occurred.

Have any limitations of the research been acknowledged?

Yes

Is the study design appropriate and does the work have academic merit?

Yes

Is the work clearly and accurately presented and does it cite the current literature?

Yes

If applicable, is the statistical analysis and its interpretation appropriate?

Not applicable

Are all the source data underlying the results available to ensure full reproducibility?

No

Are the conclusions drawn adequately supported by the results?

Yes

Are sufficient details of methods and analysis provided to allow replication by others?

Partly

Reviewer Expertise:

NA

I confirm that I have read this submission and believe that I have an appropriate level of expertise to confirm that it is of an acceptable scientific standard, however I have significant reservations, as outlined above.

# Response

**Published:** 2023-07-01

**Authors:** Mary Ellen J. Goldhamer, MD MPH

**Affiliations:** Department of Medicine, Harvard Medical School and Massachusetts General Hospital, Boston, Massachusetts, USA

Dear Dr. Winston, Thank you for your timely and careful review of our manuscript. Below, please find our point-by-point responses to your queries. We note where your suggestions were incorporated into the “Version 2” manuscript and hope you will re-review the accompanying “Version 2” manuscript with these important updates. We anticipate the updated “Version 2” manuscript will be circulated for your review in the next 1-2 weeks by the editorial staff.  We thank you in advance for reconsidering the paper for publication. Sincerely, Jean E. Klig, MD and Mary Ellen Goldhamer, MD MPH (on behalf of our author group) **Reviewer query #1:** **This study appears to be clearly presented, cites current literature, and showcases an interesting and valuable approach to remediation. The authors draw on a number of important principles, including a blend of both group and individual approaches, using a strength based appreciative enquiry coaching method that helpfully avoids a deficit model and thus appears to reduce the stigma of remediation.** **Author response #1:** Thank you.

**Reviewer query #2: The paper could be improved with clarification of a few points:**

**You state that students were identified as ‘at risk’ based on OSCE performance of ‘below passing’. What exactly does this entail? You seem to be reporting results relative to the class mean. Are you saying everyone below the mean is not passing? Obviously not, but it is unclear.** **Author response #2** **: **Thank you for this important query. We have updated the manuscript to include specific information relevant to the identification of both student cohorts in the pilot.  The following paragraph was added to the Methods section, paragraph 2: “Students were identified as ‘at risk’ based on low OSCE performance and/or concerns on narrative evaluations. A ‘below passing’ OSCE performance was the lowest fifteenth percent of scores for the clerkship cohort (cohort 1). Two students in the clerkship cohort 1 with OSCE scores at the mean on pilot entry were identified based on narrative evaluations. [Table 1] The post-clerkship cohort (cohort two) was identified based scores below the passing threshold score of 68% on the HMS summative graduation OSCE. The 68% total passing score for the HMS summative graduation OSCE was calculated using a modified Hofstee method [Burr, S.A., Whittle, J., Fairclough, L.C. *et al*.] that incorporates historical data of students’ performance and a faculty mean score based on expectations for minimum and maximum passing scores and percentages.” An earlier reference to Table 1 has been added. Reference: Burr, S.A., Whittle, J., Fairclough, L.C. *et al.* Modifying Hofstee standard setting for assessments that vary in difficulty, and to determine boundaries for different levels of achievement. *BMC Med Educ* **16**, 34 (2016). https://doi.org/10.1186/s12909-016-0555-y **Reviewer query #3:** **It would be helpful to offer some rationale for your choice of coaching model: why strengths-based framework, as opposed to other options?** **Author response #3** **: **The following statement was added to the Methods section, paragraph 4: *“These [appreciative inquiry methods and a strengths-based coaching] frameworks were chosen over models of behavior change to promote self-reflection, self-directed learning, and co-creation of learning goals. This framework aligned with the pilot’s CS (clinical skills) coaching goals which aimed to engage students and promote lifelong learning strategies.”* Further, in the Discussion and conclusions section, paragraph 2, we include a include a paragraph on how this current pilot builds on previous coaching pilots.  The updated manuscript includes the following: “ *This coaching pilot builds on existing work that addresses clinical skills remediation (Parsons A, Warburton, K et al), notably for students identified as ‘at risk’ based on a multi-station summative OSCE (Chang, Chou, Hauer) or for graduate medical education learners (Guerrasio, Brooks, Rumack et al), by specifically designing coaching-based activities for two levels of undergraduate learners (clerkship students and post-clerkship students).  It aims to further the learner-driven aspects of prior studies, in this case allowing targeted student remediation (Boileau, St-Onge, Audetat) to be guided by faculty through a dynamic and iterative co-creation process (Steinert Y). The pilot contributes towards a standardized approach to active remediation, with trained faculty coaches to support struggling learners.* *Key aspects of a targeted remediation (Boileau,* *St-Onge, Audetat* *) were embedded within the coaching pilot, whereby the individual student performance, stated goals, and engagement in individual and group coaching sessions shaped a clear process that was locally available and integral to the standard Harvard Medical School curriculum*.”

1. Parsons A, Warburton K, Martindale J, et al.: Characterization of Clinical Skills Remediation: A National Survey of Medical Schools. *Southern Medical Journal. *2022; 115 (3): 202-207 | Publisher Full Text
2. Chang, A., Chou, C.L. and Hauer, K.E. (2008), Clinical skills remedial training for medical students. Medical Education, 42: 1118-1119. https://doi.org/10.1111/j.1365-2923.2008.03191.x
3. Guerrasio, Jeannette MD; Brooks, Elizabeth PhD; Rumack, Carol M. MD; Aagaard, Eva M. MD. The Evolution of Resident Remedial Teaching at One Institution. Academic Medicine 94(12):p 1891-1894, December 2019. | DOI: 10.1097/ACM.0000000000002894
4. Boileau E, St-Onge C, Audétat M-C. Is there a way for clinical teachers to assist struggling learners? A synthetic review of the literature. *Adv Med Educ Pract*. 2017;8:89-97, https://doi.org/10.2147/AMEP.S123410
5. Yvonne Steinert (2013) The “problem” learner: Whose problem is it? AMEE Guide No. 76, Medical Teacher, 35:4, e1035-e1045, DOI: 10.3109/0142159X.2013.774082

 
**Reviewer query** **#4: You state that “Each student attended a three-hour required group coaching session”, but figure 2 seems to be imply multiple group sessions. Can you clarify this discrepancy?** **Author response #4** :  To clarify the differences between the two cohorts we note in the Methods section, paragraph 6, “[Clerkship] Students attended three three-hour required group coaching sessions and could opt for individual coaching during clerkships.”  Figure 2: Early clerkship students (cohort 1) attended three 3-hour group sessions that covered history taking (session 1, 3 hours), hypothesis-driven physical exam (session 2, 3 hours), and clinical reasoning and oral presentation (session 3, 3 hours). To clarify the “post-clerkship” cohort 2 students; the “Methods” section, paragraph 7 notes: “Manuscript Version 2” was updated to include the following: “Each post-clerkship student attended one three-hour required group coaching session with optional individual coaching.” (The word “one” replaced “a” in reference to the group coaching session.) The original Figure 2 stated “Cohort 2 Group coaching: 3-hour session, held twice”.  The authors would like to clarify that the same session was held twice to ensure attendance. For clarity, the version 2 manuscript includes an updated Figure 2 which states: “ *Cohort 2: Group Coaching: 3-hour session”. *Thank you for noting the need for this clarification. **Reviewer query #5:** What was the rationale for the decision to make the individual sessions optional? **Author response #5** : The following statement was added to the “Methods” section, paragraph 6: *“* *Individual sessions were optional to avoid overburdening students and to gauge student engagement in co-creating their learning. ”* **Reviewer query #6** :  There were “frequent check-in meetings”. How many of these per student? How frequent? Optional or required? Individual or group? **Author response #6** : The following statement was added to the “Results” section, paragraph 1: *“* *Optional individual clerkship student (cohort 1) check-ins occurred in person, over email, and by phone and were offered monthly, with a range of 0-4 check-in meetings per clerkship student.”* **Reviewer query #7:** **Figure 5 suggests 14% of cohort one had pre-clerkship OSCE performance at or above class mean, and yet earlier you stated that entry to the pilot required a below passing score. How is this discrepancy explained? Or am I misunderstanding the data, in which case more clarity may be needed, perhaps** **Author response #7** : Thank you for this query. Clerkship students, cohort 1 were identified by below passing pre-clerkship OSCE scores and/or performance evaluations indicating concern(s) in the area of clinical skills. The revised “Manuscript Version 2” Methods section, paragraph 6, statement **has been revised for clarification from: **“ *The clerkship student cohort (cohort one) was identified based on below passing pre-clerkship OSCE scores **and** evaluations*” **to now state:** “ *Students were identified as ‘at risk’ based on low OSCE performance and/or concerns on narrative evaluations. A ‘below passing’ OSCE performance was the lowest fifteenth percent of scores for the clerkship cohort (cohort 1). Two students in the clerkship cohort 1 with OSCE scores at the mean on pilot entry were identified based on narrative evaluations. [Table 1] The post-clerkship cohort (cohort two) was identified based scores below the passing threshold score of 68% on the HMS summative graduation OSCE.* *The 68% total passing score for the HMS summative graduation OSCE was calculated using a modified Hofstee method [* *Burr, S.A., Whittle, J., Fairclough, L.C.* *et al* *.] that incorporates historical data of students’ performance and a faculty mean score based on expectations for minimum and maximum passing scores and percentages*.” An earlier reference to Table 1 has been added. Reference: Burr, S.A., Whittle, J., Fairclough, L.C. *et al.* Modifying Hofstee standard setting for assessments that vary in difficulty, and to determine boundaries for different levels of achievement. *BMC Med Educ* **16**, 34 (2016). https://doi.org/10.1186/s12909-016-0555-y **Reviewer query #8:** **The sustained improvement beyond the pilot is impressive – readers might be interested to hear why you think this occurred.** **Author response #8** **:** Thank you. The following sentence was added to the “Discussion” section, paragraph 1: “ *The skills of reflective practice and self-directed learning were iteratively emphasized through appreciative inquiry and strengths-based coaching strategies, and it is likely the students applied these strategies to other aspects of their learning, resulting in sustained improvement in OSCE scores over two years* *.”*
